# Process Enhancement of Calcium Looping through Combined Mechanical and Chemical Sorbent Reactivation

**DOI:** 10.1002/cssc.202500835

**Published:** 2025-08-13

**Authors:** Dominik Groh, Pawel Chmielniak, Christopher W. Jones, Carsten Sievers

**Affiliations:** ^1^ School of Chemical & Biomolecular Engineering Georgia Institute of Technology 311 Ferst Dr Atlanta GA 30332 USA; ^2^ Department of Chemical and Bioengineering Friedrich‐Alexander Universität Erlangen‐Nürnberg Cauerstr. 4 91058 Erlangen Germany

**Keywords:** calcium looping, carbon capture, industrial chemistry, porous materials, sorbent reactivation

## Abstract

This study tackles sorbent deactivation in the calcium looping process by combining mechanical and chemical reactivation via wet planetary milling. Consequently, sorbent performance is enhanced via leveraging reactivation agents and transient microenvironments simultaneously. Reactivation is systematically applied after each calcination–carbonation cycle, a process termed remilling. Remilled sorbents exhibit 2–3 times higher CO_2_ capture capacity than untreated ones over the first 10 cycles. This improvement is attributed to Ca(OH)_2_ formation during milling and the mechanical disruption of sintered particles, which both re‐expose previously inaccessible CaO domains. A supporting semiempirical model is implemented and aligns with these findings. The model highlights the ongoing creation and closure of small‐scale pores. The detailed physicochemical characterization of the sorbent properties reveals the dominant influence of the milling regime as well as de‐ and reactivation mechanisms. The observations underline an increased surface area, formation of small‐scale pores, higher CaO conversion, and less deactivation. This suggests that high space‐time yield within the overall process is obtained, and sorbent replacement rates are lowered. A large‐scale scenario comparison of energy demand for remilling and sorbent replacement highlights the potential and boundaries of the suggested process adaptation.

## Graphical abstract

Remilling—mechanical and chemical reactivation via wet milling after each cycle in the calcium looping process—boosts CO_2_ capture by 2–3 times, enhances sorbent properties, and reduces deactivation and replacement rates, as confirmed by physicochemical characterization and supported by a semiempirical model.

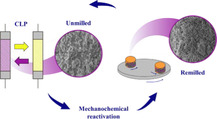

## 1 Introduction

Within the last decades, the carbon dioxide (CO_2_) content of the atmosphere has increased from 280 ppm (preindustrial level) to around 420 ppm due to anthropogenic emissions, making it one of the most significant greenhouse gases.^[^ ^1^ ^]^ This open carbon “cycle” results in excessive atmospheric CO_2_, and the consequences are wide‐ranging: Examples such as the increase in the global mean surface temperature, ocean acidity rise, and glacier thickness loss show the urgency for action.^[^ ^2^ ^]^ Because humanity still relies on fossil fuels for power generation, transport, and production of goods while growing the portfolio of renewable energy,^[^ ^3^, ^4^ ^]^ closing the carbon cycle is one of the most important global engineering challenges. To this end, carbon capture, utilization, and storage have been investigated as an approach to remove CO_2_ from flue gas emitters.^[^ ^5^, ^6^, ^7^ ^]^ Here, the idea is to capture CO_2_, e.g., with the use of a sorbent and store this CO_2_ for later use or sequestration. An auspicious approach is the calcium looping process (CLP), which has been successfully implemented in large‐scale applications.^[^ ^8^, ^9^ ^]^ This high‐temperature process uses the reversible reaction of calcium oxide (CaO) with CO_2_ to form calcium carbonate (CaCO_3_) (Equation (1)).^[^ ^10^ ^]^ The CLP is currently used as a postcombustion approach and appeals due to its initially high adsorption capacity, low cost, effective integration within high‐temperature plants, and wide material availability.

(1) $${\text{CaO}}_{\left(s\right)}{\text{ + CO}}_{2\left(g\right)}\;⇌{\text{ CaCO}}_{3\left(s\right)}{\text{ ΔH}}_{298}^{0}=\text{‐178 kJ}\;{\text{mol }}^{\text{‐1}}$$

The CLP involves two main steps: carbonation (CO_2_ uptake) and calcination (CO_2_ release). During carbonation, CO_2_‐rich postcombustion gas (≈15 vol.‐% CO_2_) is fed into a circulating fluidized bed system at temperatures of ≈650 °C, reacting with CaO to form CaCO_3_. The subsequent calcination step releases the chemically bound CO_2_. To process or store this released CO_2_, the calciner must be operated at a high CO_2_ partial pressure (≈0.15 barg). Therefore, calcination requires high temperatures (T ≈ 900 °C).^[^ ^11^, ^12^, ^13^ ^]^ The main challenge in implementing the CLP is the low thermal stability of the sorbent (*T*_Tamman, CaCO3_ =533 °C) and harsh process conditions, which lead to cycling‐induced deactivation as well as sintering of CaO particles. The result is a rapid drop in the CO_2_ capture efficiency within the first few cycles.^[^ ^14^, ^15^ ^]^ Consequently, the sorbent material must be replaced periodically to compensate for the formation of a firm backbone of less reactive sorbent with lower surface area, decreased pore volume, and higher diffusional resistance.^[^ ^16^, ^17^, ^18^ ^]^ Therefore, a scalable regeneration strategy that either decelerates deactivation or even reactivates spent sorbent would be a significant process improvement.

The CLP has been widely studied, and various possible enhancements have been explored. For example, chemical reactivation of sorbents (e.g., by hydration) has been demonstrated to be effective on a laboratory scale, with drawbacks when it comes to attrition, space‐time yield, and energy consumption.^[^ ^19^, ^20^ ^]^ The use of dopants,^[^ ^21^, ^22^, ^23^ ^]^ structural stabilization,^[^ ^24^ ^]^ chemical pretreatment,^[^ ^25^ ^]^ polymorphic spacers,^[^ ^26^, ^27^ ^]^ or thermal pretreatment^[^ ^28^ ^]^ are options to prepare initially superior CLP sorbents. However, these methodologies require additional chemicals, generate higher costs, or result in process behavior that is difficult to predict. In contrast, using mechanical pretreatment is a cost‐effective, energy‐efficient, and easy‐to‐scale option to prepare CLP sorbents. The mechanical impacts encountered in ball‐milling can cause transient, localized hot spots, fracturing of particles, formation of defects, lowering of crystallinity, phase transformations, and a decrease of crystallite sizes.^[^ ^29^, ^30^, ^31^ ^]^ Furthermore, milling characteristics can be fine‐tuned using a solvent or dispersant for optimal lubrication, force distribution, and dispersion of particles in a liquid‐assisted or wet‐milled environment.

Most attempts at mechanochemical sorbent activation have only focused on maximizing the CO_2_ uptake. These studies have investigated the influence of initial sorbent milling on the cyclic CO_2_ uptake behavior within the CLP and have shown that the effectiveness of such a single sorbent preparation step is dependent on the CaO precursor (limestone, dolomite, pure CaCO_3_, and commercial CaO),^[^ ^32^, ^33^ ^]^ the cycling conditions,^[^ ^34^, ^35^, ^36^, ^37^ ^]^ and the type of milling (vibratory/planetary/combined, wet/dry).^[^ ^36^, ^38^ ^]^ Using appropriate milling conditions can yield a highly effective CLP sorbent. On the other hand, nonoptimized milling can result in the opposite. Previous studies determined the consequences of specific milling parameters. The calcination kinetics can be accelerated through defects and lattice stresses in ball‐milled low‐crystalline sorbents,^[^ ^33^, ^39^, ^40^, ^41^ ^]^ and decreased particle dimensions can enhance the reactivity of CaO.^[^ ^34^ ^]^ Change in crystallite size during cycling is also important and keeping crystallites small can promote lattice diffusion.^[^ ^36^ ^]^ At the same time, cold‐welding phenomena through ball‐milling occur in dry‐grinding systems^[^ ^36^ ^]^ and a milled CLP sorbent can occasionally deactivate faster than an untreated powder.^[^ ^42^ ^]^ Although it has been demonstrated that reactivation of sintered sorbent is possible, studies investigating mechanical reactivation were restricted to only one single reactivation treatment. At the same time, the experimental conditions were unfeasible for industrial processes, because calcination conditions were mild, and milling times up to 24 h were proposed.^[^ ^34^, ^43^ ^]^

The present work examines milling as a reactivation strategy for spent sorbents after each calcination–carbonation cycle. It enhances CLP sorbent performance via rapid mechanochemical reactivation, demonstrating sustained improvements over 10+ cycles. A fast preparation method for CLP precursors, optimizing multicyclic CaO activity, is identified and compared to reference samples. Key reaction parameters validate the efficiency of this fast and feasible approach. A semiempirical model and the physicochemical analysis uncovers the reactivation mechanism, its CLP suitability, and its impact on sorbent carbon capture efficiency. Energy demand scenario calculations add an industrial perspective and evaluate feasibility on a large‐scale.

## 2 Results and Discussion

### 2.1 Characterization of the Fresh Sorbent and Baseline Performance Testing

X‐ray diffraction (XRD) analysis of the pristine CaCO_3_ confirmed that it consisted of calcite with reflections at 2*θ* = 23.0°, 29.4°, 31.4°, 35.9°, 39.4°, 43.2°, 47.1°, 47.5°, 48.5°, 56.6°, and 57.4° with high crystallinity. The crystallite size based on the [1 0 4] reflection was 55.4 nm. The apparent Brunauer–Emmett–Teller (BET) surface area of the uncalcined material was 0.66 m^2^ g^−1^. After calcination for 1 h at 950 °C, this value rose to 25.1 m^2^ g^−1^. After 10 cycles of calcium looping, the apparent surface area of the calcined sorbent dropped to 4.2 m^2^ g^−1^. The Barrett–Joyner–Halenda (BJH) pore size of the calcined sample showed a unimodal distribution with meso‐ to macropores and an average pore diameter of ≈36 nm. After 10 cycles, the average pore diameter shifted to ≈29 nm, confirming ongoing aging and capacity loss of the sorbent. At the same time, the pore volume decreased by 87%. Laser diffraction analysis (LDA) determined that the particle size distribution (PSD) was nearly unimodal with d_10_ = 4.2 μm, d_50 _= 18.4 μm, and d_90_ = 45.2 μm. Thermogravimetric analysis (TGA) analysis of the untreated powder revealed a mass loss of 44.6 wt.‐% during the first calcination.

### 2.2 Overcoming Sorbent Deactivation by Remilling

**Figure 1:**
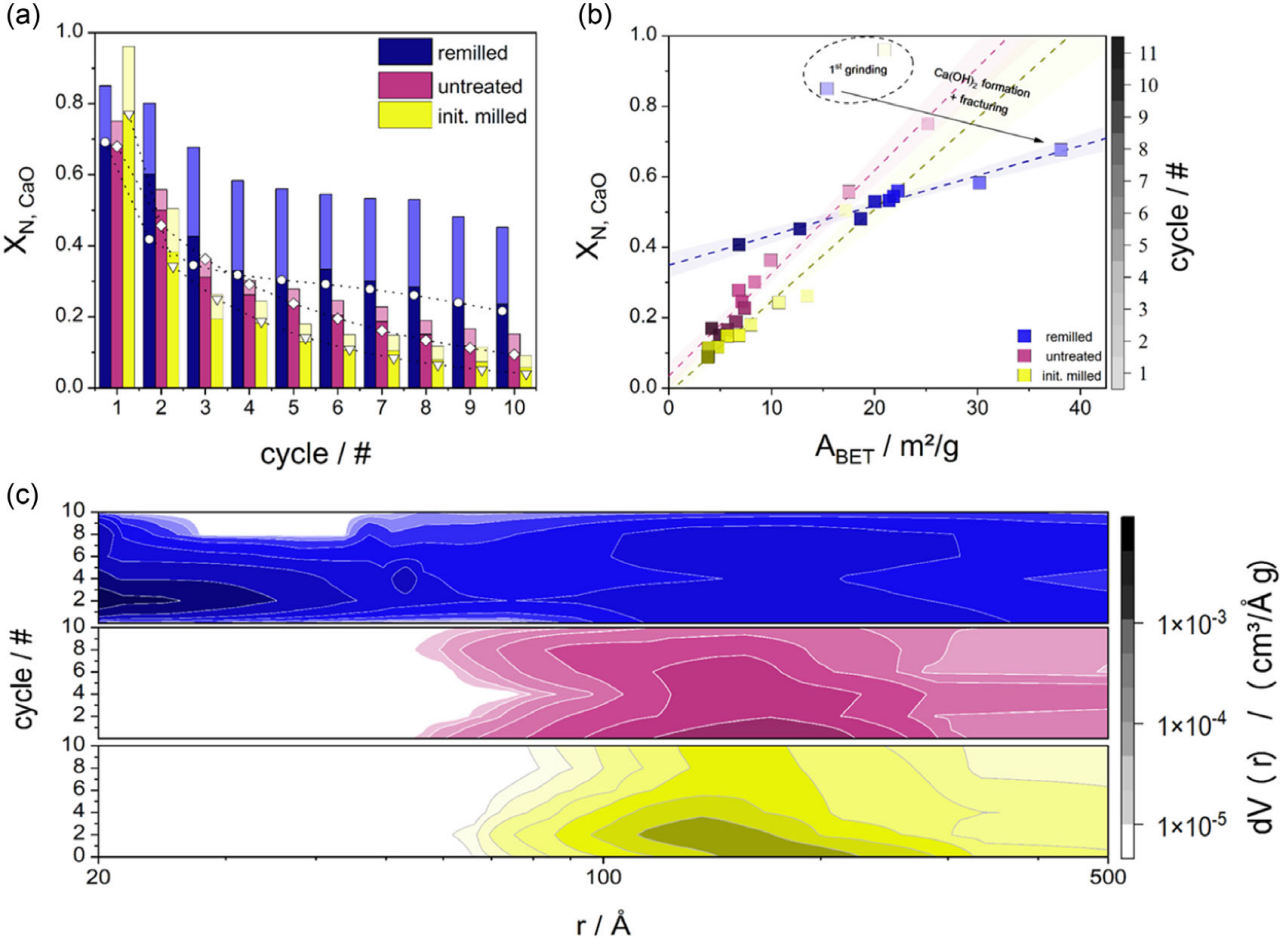
a) Multicycle CaO conversion of remilled, untreated, and initially milled samples. Values were calculated from TGA measurements after passing N‐1 cycles within the fixed bed reactor setup (1 h of calcination at 950 °C and 4 h of carbonation at 650 °C). Dark: FRP, Bright: SDP, Model fit: Circles – remilled, squares – untreated, triangles – initially milled. b) BET surface area analysis for remilled, initially milled, and untreated CLP sorbent. c) BJH pore size distributions for remilled (blue), untreated (red), and initially milled (yellow) CLP sorbent. The microcrack‐induced formation of small pores <40 nm is apparent.

**Figure 2:**
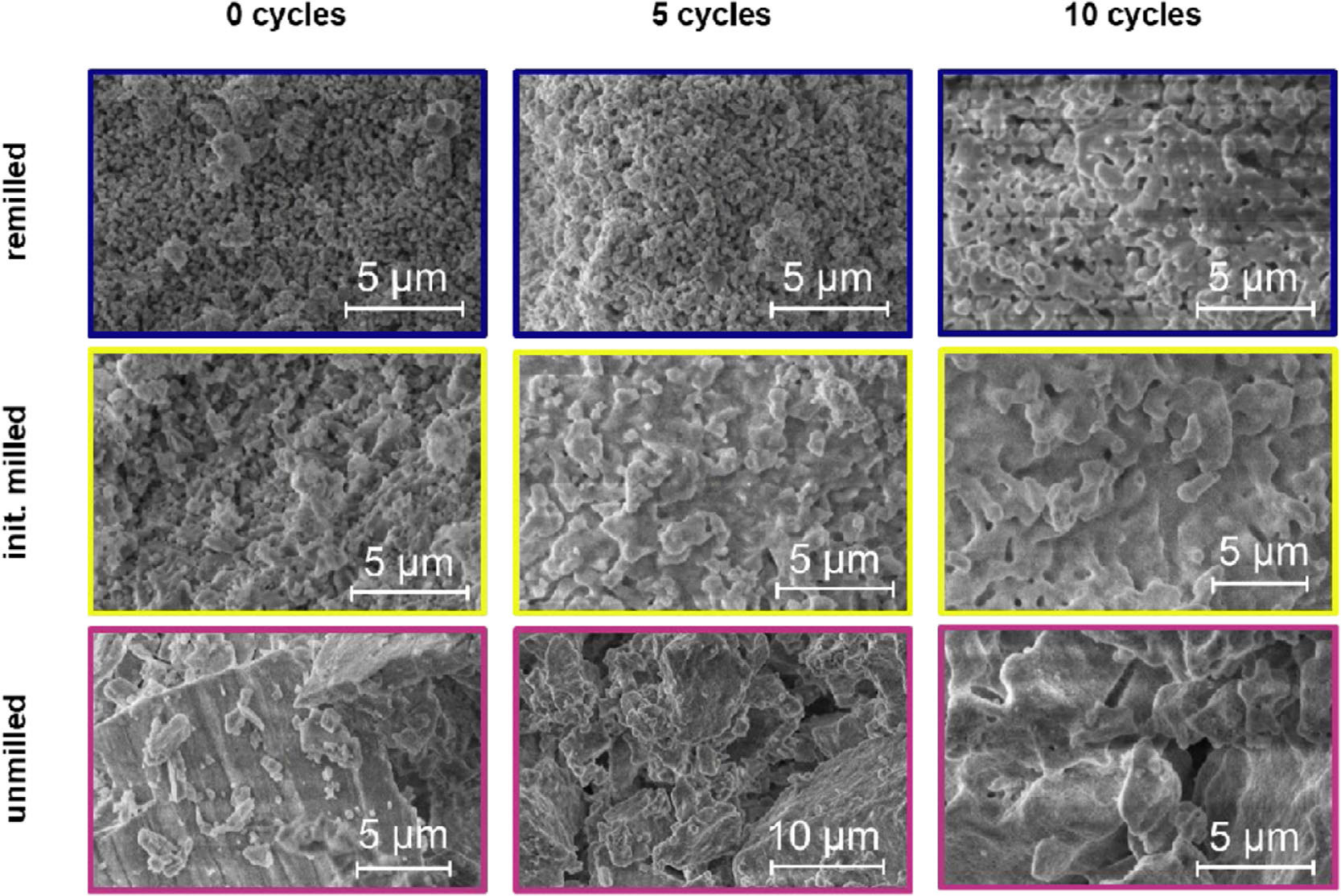
SEM micrographs of remilled (blue, top row), initially milled (yellow, middle row), and untreated (red, bottom row) CLP sorbent. Sintering over time occurs for all samples, but is most dominant for the initially milled sorbent.

The CO_2_ uptake capacity of the pelletized, fresh reference material decayed exponentially with increasing CLP cycle number. Starting at ≈75% conversion with the main contribution from fast reaction‐controlled phase (FRP) (≈70%, indicated as dark area in all figures), a fast reduction to ≈15% conversion with increased slow diffusion‐controlled phase (SDP) contribution (≈5%, indicated as bright area) occurred within the first 10 cycles (**Figure** **1**a). Especially in the beginning, a loss of almost half of the initial CO_2_ uptake capacity was apparent owing to the fast loss of reactive surface area because of aging. This was reflected by a steep decrease in surface area from 25.1 m^2^ g^−1^ (cycle #1) to 9.9 m^2^ g^−1^ (cycle #3) and 4.2 m^2^ g^−1^ (cycle #11) (Figure 1b). Similarly, the mesopore volume and average pore size decreased significantly (Figure 1c). Accordingly, the sorbent morphology reflected sintering on a micrometer level (**Figure** **2**). Within the 10 cycles investigated, neck formation after five cycles was apparent and intensified to the end of the experiment. Consequently, the gradual elimination of pores within the micrographs was visible, resulting in a solid and densified microstructure.

To mitigate these issues, the remilling of sintered CaO should create a highly effective CLP sorbent^[^ ^32^, ^34^, ^36^, ^38^, ^39^, ^44^ ^]^ (and subsection “Physicochemical Properties of the Sorbents and Consequences for the Remilling Reactivation Pathway”), where deactivation is reversed cyclically. Initial milling, while possibly enhancing the CLP, still yields a material that suffers from fast deactivation due to sintering and carbonation/calcination cycles. Milling the sorbent after every cycle of calcination and carbonation, as an advanced process strategy, was expected to eliminate these influences directly.

Figure 1a shows the conversion of CaO as a function of cycle number for the three investigated sorbent types, as well as the model fit, which includes the sintering constant *k*_p_, the milling rate constant *k*_m_, and the rate constant for Ca(OH)_2_ formation *k*_Ca(OH)2_ as the main parameters. The implemented model fits the FRP data well after the first three cycles. Even though new model parameters (**Table** **1**) were introduced for remilled material, the experimental data were underestimated in the first three cycles. This issue was more pronounced for the remilled samples than the references because the exposure of CaO domains to water during the remilling process is not entirely captured by the model. At the same time, a high value of the sintering constant (*k*_p_) demonstrated a dynamic system, in which the effect of sintering is offset by fracturing. The main cause is a localized density change due to the reaction of CaO with H_2_O, which results in inhomogeneous swelling and, therefore, the creation of microchannels within the structure. The sintering constant of the two reference samples was identical. This underlines the validity of the presented modeling approach and might even suggest extrapolative capabilities. Taking a deeper look into the data, the total conversion of CaO of an initially milled sample started at a high value of ≈95% and decayed exponentially. After only two cycles, the conversion of CaO of an initially milled sample remained below an untreated sample. In both cases, the conversion was dominated by the FRP (≈80% of total conversion), whereas the SDP contribution was low (<20%). A remilled sample also started at a high total conversion (≈85%). Discrepancies to the initially milled sorbent within the first cycle are attributed to manual compaction, fluctuating humidity in the laboratory, and systematic errors that become especially significant when investigating the first CO_2_ uptake. Nevertheless, for remilled material, unlike for the other samples, its CO_2_ uptake plateaued at a high CaO conversion of 50%–60% (average) for 10 subsequent cycles. Here, SDP and FRP contributed roughly 50% each to the total conversion at steady state. Although a 30‐min mechanochemical treatment after every cycle was not sufficient to fully regain the initial capacity of the sorbent, it still managed to multiply it compared to both an untreated and an initially milled sample (Figure 1a).

**Table 1:** Fitting parameters of Equation ((4), (5), (6), (7), (8), (9), (10), (11))–(12) to the data shown in Figure 1a.

Treatment	*ε*_0_ [m^3^_void_ m^−^^3^]	k_p_ [min^−1^]	k_m_ [min^−1^]	*k*_Ca(OH)2_ [min^−1^]	*V*_c_ [cm^3^ mol^−1^]	R^2^ [−]	*ω* ^2^ [−]
Remilling	0.4	0.8	0.2	0.2	3.8	0.988	0.946
Untreated	0.4	0.3	–	–	–	0.979	0.953
Initial	0.4	0.3	0.1	–	–	0.985	0.965

The remilling strategy was highly effective at extending the lifetime of the sorbent. While the untreated and initially milled CaO deactivated rapidly, remilled CaO remained an excellent sorbent during calcination–carbonation cycles. This difference became particularly evident between cycles #4 and #8. During this stage, an almost stable performance of the sorbent was achieved. As seen in previous studies, the loss in CO_2_ uptake capacity is highest during the first few cycles, even for initially milled CLP sorbents.^[^ ^32^, ^39^, ^44^ ^]^ Therefore, it is not surprising that some capacity was lost in cycles #1–#3. It is assumed that the creation of surface area, porosity, and crystal defects through remilling is not high enough to entirely outperform the elimination of sorbent capacity by sintering during these first cycles. The extended carbonation–calcination cycles suggested that a steady state can be reached after a greater number of cycles and that this steady state stabilized on a higher residual conversion level than for the initially milled or untreated CaCO_3_. Interestingly, the FRP of the remilled powder in all cycles was higher than the combined conversion of FRP and SDP of the reference materials. This boost in the FRP of remilled sorbent illustrated that the effectiveness of this procedure is due to diffusion enhancement toward the open, unreacted CaO sites. This enhancement has to be caused by a structural and morphological change in the sorbent material.

There are two nuances when comparing the initially milled sorbent sample to the untreated and remilled samples. Both the remilled and initially milled sorbents showed improved performance during the first cycle. However, the conversion of the initially milled sorbent decreased within two cycles and ultimately fell below the conversion level of the untreated sorbent. The slightly faster deactivation of the initially milled sample compared to the untreated reference can be explained through process differences: All samples were pelletized before carbonation, which is a key difference from most prior studies. The initially milled and remilled samples had a higher accessible surface area, which explains the superior CO_2_ capacity of both during the first cycle. Sorbent pellets were used to ensure that all samples experienced the same process conditions, i.e., similar CO_2_ flux, comparable pressure drop, and no particle size effects. Unlike powder beds, pelletized sorbents mitigate any influence of shifted PSDs seen in previous studies. The initially milled sorbent had a higher initial conversion but deactivated faster because of sintering. At the calcination temperature of 950 °C, smaller crystals tend to sinter faster. This is referred to as “melting point depression”^[^ ^24^ ^]^ and was seen in the fast decline for the initially milled sorbent. For the untreated sample, no crystal size reduction occurred, whereas the milling was at least partially reducing crystallite sizes. For the remilled sorbent, the crystallite size should be less relevant since the treatment is repeated after every cycle.

Because sorbent activity was linked to diffusion limitations, its surface area was expected to be a good predictor for CaO conversion. The BET surface area of the untreated and initially milled sorbents started at ≈20–25 m^2^ g^−1^ and rapidly decreased to 5–10 m^2^ g^−1^ within four cycles (Figure 1b). In contrast, the BET surface area of the remilled sorbent started at 15 m^2^ g^−1^, rose to almost 40 m^2^ g^−1^ within two cycles, and plateaued at 20 m^2^ g^−1^ for five subsequent cycles before eventually dropping to ≈7 m^2^ g^−1^. These experiments underline that the surface area and CaO conversion are highly correlated (Figure 1b). The BET surface area of the untreated and initially milled sorbent decayed exponentially, and the CaO conversion followed the same decay behavior. Both initially milled and unmilled sorbents seemed to approach small residual conversion when extrapolating to a high number of cycles. In contrast, the remilled sorbent follows a different slope with a residual conversion exceeding that of the reference sorbents more than twofold. These differing slopes indicated that while surface area declines over time for all sorbents, the remilling procedure significantly slows this decline. This leads to a sustained advantage by maintaining higher surface area and conversion levels for longer. In addition, the loss in capacity for remilled sorbent needs to be discussed. This behavior might be a cause of ongoing sintering that is not restored by milling anymore. The carbonation reaction inside the CLP quickly becomes diffusion‐limited. Remilling addresses this issue by increasing the surface area, which enhances accessibility to unconverted CaO sites and facilitates further reaction.

The increase in surface area was not only because of the mechanochemical rupturing of particles during remilling but also due to Ca(OH)_2_ formation. This is because the sorbent was milled wet (Section 4 ); therefore, most CaO was converted into Ca(OH)_2_. The formation of Ca(OH)_2_ after the second cycle was confirmed by XRD and TGA (Figure S1, Supporting Information). Ca(OH)_2_ is less dense than CaO. This density change results in swelling of the structure and causes microscopic cracks and the formation of channels inside the sorbent.^[^ ^20^ ^]^ Therefore, a combined mechanical and chemical reactivation for the remilled sorbent was achieved. Such an effect was absent for both the untreated and the initially milled sorbent. As already briefly mentioned, the plateau in surface area between cycles 4 and 8 is particularly interesting. As soon as the smaller pores collapse, the internal surface area becomes inaccessible, and only the external surface remains available for CO_2_. The slight decline toward the end was likely due to remaining densification on much smaller and slower scales. Lastly, milling enhances the conversion of CaO to Ca(OH)_2_ compared to soaking by accelerating reaction kinetics through mixing, increased substrate contact, and creation of transient reactive microenvironments. All these phenomena contribute to the reactivation (Section 2.4). In addition, the high operational temperature during the CLP results in the decomposition of Ca(OH)_2_ to form water that passes through the reactor as steam. Steam is known for its catalytic effect in the CLP during calcination, which likely has an enhancing effect on the CLP.^[^ ^45^ ^]^ Additionally, pressure buildup from trapped water bubbles can create additional cracks and accessible CaO sites as they seek to escape the heated system.^[^ ^46^ ^]^ The trends of the BET surface area were qualitatively confirmed by scanning electron microscopy (SEM) micrographs (Figure 2). Sintering was observed for both milled samples, but it was more pronounced for the initially milled sorbent. The well‐known calcite structure for the untreated material remained intact even with compaction through pelletization (Figure S2, Supporting Inofmration).

The SEM analysis agreed qualitatively with the pore size distributions determined by N_2_ physisorption (Figure 1c). No pores with a radius <5 nm were observed for the untreated and initially milled sorbent during any of the cycles. Additionally, the pore sizes demonstrated ongoing deactivation of the sorbent and steadily shifted to lower values of dV(r) for later cycles. The same observations of loss in pore volume were made for remilled sorbent, with the difference of appearing micropores after the first cycle. This enhanced the sorbent's pore volume and reduced deactivation massively. Evaluation of the crystallite size for all the samples did not yield a coherent trend, and it is assumed that the influence of the crystallite size is reduced with multiple cycles (Figure S3, Supporting Information). Within this work, the influence of material porosity and the possibility of reaching unconverted CaO through pore diffusion is suggested to be more relevant than grain boundary or lattice diffusion effects.

In summary, remilling was shown to be highly effective and promises better use of the sorbent within the process. This is a major improvement since milling is nonenergy‐intensive.

### 2.3 Large‐Scale Energy Demand of Remilling Compared to Sorbent Replacement

**Figure 3:**
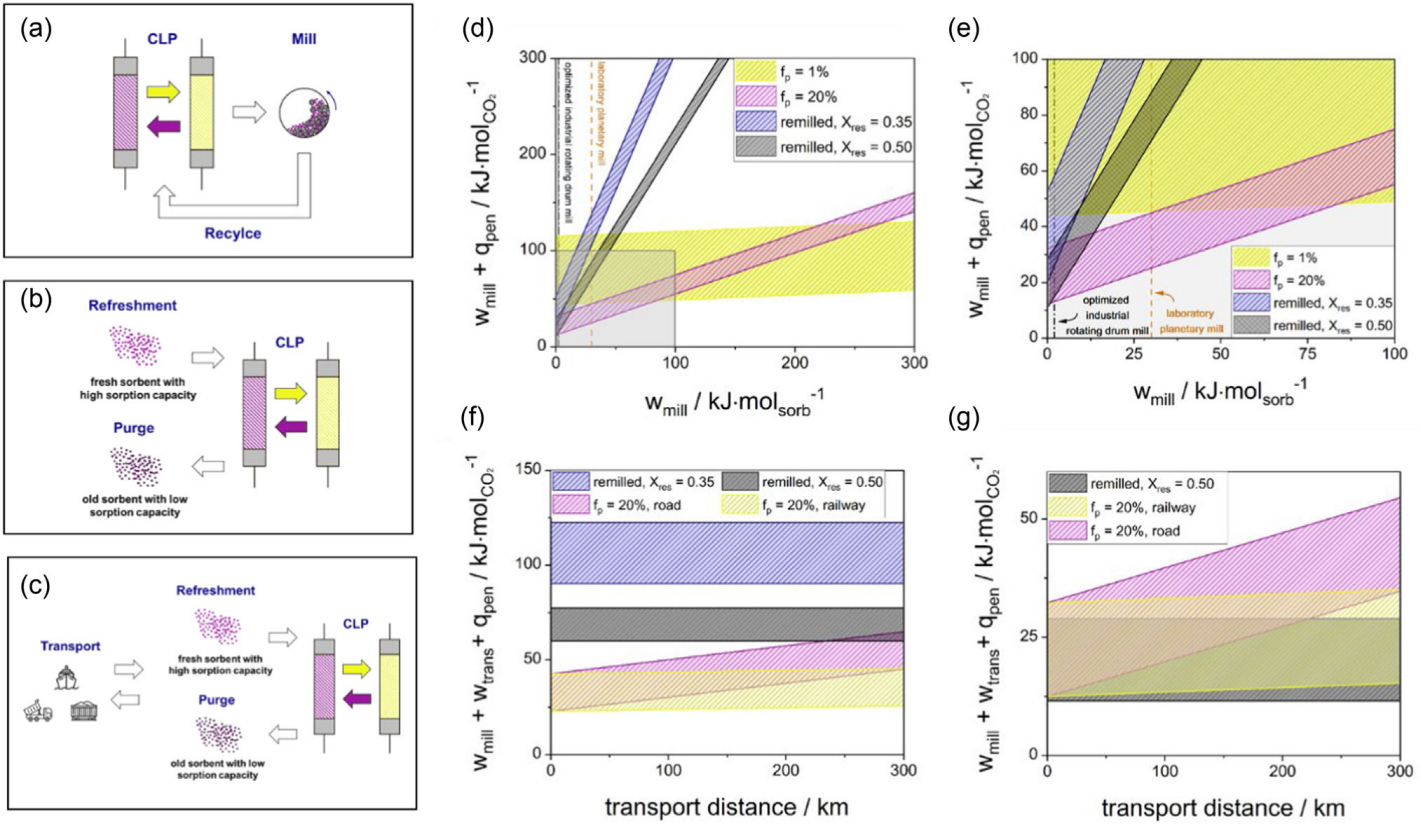
a–c) Overview for the three investigated scenarios and d–g) energy demand comparison for selected scenario results. (a) Scenario (i): Remilling‐enhanced CLP. (b) Regular CLP with sorbent refreshment. (c) Regular CLP with sorbent refreshment, including transport of fresh sorbent to the process site. (d) Combined energy penalty from milling and unused sorbent heating as a function of the specific milling energy demand. The shaded areas highlight calcination temperatures ranging from 850 °C to 1150 °C. Sorbent refreshment rates were chosen to be 1% and 20%, respectively. For remilling, a pessimistic scenario with *X*_res_ = 0.35 and an optimistic scenario with *X*_res_ = 0.5 frame possible boundaries of the enhanced CLP. (e) Zoom‐in of panel (d) providing details for the region of high milling efficiency, which becomes especially relevant at an industrial scale. (f) Additional impact of sorbent transport in the case of sorbent refreshment. Energy demand of milling was chosen to be 24.6 kJ·mol_sorbent_^−1^. Calcination temperatures range from 850 °C to 1150 °C and frame the shaded areas. Sorbent refreshment rates are set to 20% for both cases with transport on road or railway. (g) Same scenario simulation as in (f) with the difference of efficient, large‐scale milling (*W*_mill_ = 0.4 kJ mol_sorbent_^−1^).

To evaluate whether remilling could serve as a viable adaptation for an industrial‐scale CLP, three sorbent management scenarios were assessed (**Figure** **3**a–c and subsection “Energy Demand Comparison”). The remilling‐enhanced CLP configuration offers the key advantage of reducing material exchange, in contrast to the replacement scenarios (ii) and (iii), which require continuous purging and replacement with fresh sorbent. These strategies introduce additional embodied and, in the case of scenario (iii), transportation energy penalties (Figure 3a–c). Thus, the scenarios are focusing on the energy penalties associated with milling, thermal losses from inert sorbent, and transport. The results are normalized per mole of captured CO_2_ to better reflect process performance (Figure 3d–g).

Figure 3d shows that the energy demand for milling and heating increases with milling energy. This became particularly relevant under the pessimistic assumptions in scenario (i), such as a low residual conversion of *X*_CaO_ = 0.35 for the remilled sorbent. This represents a conservative estimate. Higher conversions, which are technically feasible, shift the energy balance in favor of remilling. The optimistic scenario, which assumes a residual conversion of *X*_res_ = 0.5 for a remilled sorbent, highlights this potential of the remilling approach. Although planetary milling is energy‐intensive at the laboratory scale, it is important to recognize that industrial‐scale milling technologies (e.g., rotating drum ball mills, vertical roller mills, …) are significantly more energy‐efficient and widely used in large‐scale operations. In this context, adopting the pessimistic scenario may appear overly conservative. Nevertheless, this deliberately chosen case demonstrates that remilling remains a competitive option if milling energy efficiency is high, even under unfavorable assumptions. At higher milling energies (>50 kJ mol_sorb_^−1^), sorbent replacement with moderate purge rates (e.g., *f*_p_ = 0.2) becomes more attractive and outperforms remilling as well as lower purging rates (e.g., *f*_p_ = 0.01). However, when milling energy is reduced, particularly below 25 kJ·mol_sorb_^−1^, the remilling process route becomes increasingly favorable (Figure 3e). For comparison, the planetary mill used in this study operates at around 27 kJ_mech_·mol_sorb_^−1^, indicating that even with lab‐scale equipment, remilling approaches close to the crossover point with sorbent replacement strategies, especially if the purging rates are low.

More realistic implementation scenarios must also consider the transport energy required to supply fresh sorbent (Figure 3f–g). In a scenario where milling energy demand is high (e.g., 24.6 kJ mol_sorb_^−1^), sorbent replacement is capable of outperforming remilling even with high transport distances (Figure 3f). Under conditions of high remilling efficiency (*X*_res_ = 0.5), a crossover point with sorbent replacement for long‐distance road transport occurs at ≈250 km. If the milling step is further optimized to reflect industrial‐scale energy efficiencies (e.g., <1 kJ·mol_sorb_^−1^), remilling surpasses direct sorbent replacement across the chosen transport scenarios (Figure 3g). This advantage becomes even more pronounced when considering moderate to long transport distances, making such scenarios increasingly relevant for practical deployment. Importantly, incorporating a full life cycle assessment (LCA) would further penalize transport‐heavy strategies, as associated CO_2_ emissions from fuel combustion and logistics infrastructure would increase the overall carbon footprint of the replacement pathway. In addition, the mining of the material would further worsen this scenario. Furthermore, the results normalization per mole of CO_2_ captured disadvantages remilling under the conservative assumptions chosen within this work. However, this highlights the possibility of process improvement, possibly boosting remilling and tapping optimization potential. One last uncertainty lies in the role of water during milling. While moisture can enhance CO_2_ capture via catalytic effects or pore development (subsection “Overcoming Sorbent Deactivation by Remilling”), it also introduces energy demand for drying, which was not considered herein. The trade‐off remains unresolved and should be examined further for industrial implementation.

### 2.4 Comparison of Remilling and Sorbent Hydration

To demonstrate that the sustained performance of the remilled sorbent was not only caused by hydration of the sorbent, the sample of the remilled sorbent taken after the 10th cycle was split into three batches. One batch was not treated at all, one was soaked in water for 30 min, and the last one was ground under wet conditions for 30 min (i.e., the usual remilling procedure). TGA and XRD characterization revealed that more Ca(OH)_2_ was formed during wet milling compared to only soaking it in H_2_O (12.3 wt% compared to 7.0 wt% of initial sorbent mass, respectively) (Figure S4, Supporting Information). Furthermore, physisorption data revealed a higher surface area for the wet‐milled sorbent, even though the soaked sorbent reached values that lay in the same region (6.6 m^2^ g^−1^ compared to 6.5 m^2^ g^−1^, respectively) (**Table** **2**). The untreated sorbent showed a decline in the BET surface area (2.8 m^2^ g^−1^ compared to the ≈6.5 m^2^ g^−1^of the regenerated sorbents). A similar trend was seen for the CO_2_ uptake behavior. The wet‐milled sorbent was most effective in terms of the CaO conversion, followed by the soaked sorbent and the untreated one (41% compared to 27% and 22%, respectively) (Table 2). This finding supports the theory that milling destroyed the firm CaO backbone, which was built through sintering during the previous cycles. Thereby, encapsulated, inaccessible, and consequently inactive sorbent material can be made accessible at a newly formed surface. Soaking in water also reactivated some of the activity of the sorbent. However, the diffusion through the system and the accessibility to the CaO were not as high as for simultaneous milling and soaking. Nevertheless, soaking also introduced additional porosity through swelling while transforming CaO to Ca(OH)_2_, which was reflected by the high BET surface area. The key difference is that the introduced small‐scale pores, when only soaking in water, sinter quickly, preventing diffusion soon after the start of the experiment (Table 2).

**Table 2:** BET surface area and corresponding CaO conversion values for wet remilled, water‐soaked, and untreated sorbent after 10 cycles of carbonation and calcination.

Treatment	A_BET_/m^2^ g^−1^	X_CaO_/−	X_CaO, SDP_/−	X_CaO, FRP_/−
Remilled	6.6	0.41	0.18	0.23
Soaked	6.5	0.27	0.14	0.13
Untreated	2.8	0.22	0.12	0.10

**Figure 4:**
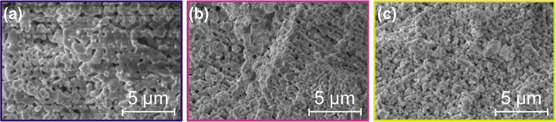
a) SEM micrographs of untreated, b) water‐soaked, and c) remilled sorbent after 10 cycles of remilled calcination‐carbonation.

Another reason for enhanced sorbent performance through wet milling was illustrated by SEM analysis. The micrographs showed a less dense morphology for the wet‐milled sample compared to the untreated or soaked sorbent (**Figure** **4**). Unlike soaking in water, wet remilling can be completed more quickly. Thus, the process can be scaled up more easily, and thereby offers a suitable way to avoid the extensive replacement of CaO with fresh sorbent.

### 2.5 Physicochemical Properties of the Sorbents and Consequences for the Remilling Reactivation Pathway

**Figure 5:**
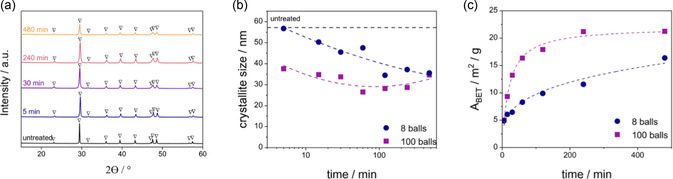
a) XRD patterns of planetary wet‐milled CaCO_3_ (100 stainless‐steel balls ‐ 3/16”) with varying duration (∇: calcite). b) Resulting crystallite size evolution for two sizes (3/16”, 7/16”) + numbers (8, 100) of grinding bodies. c) BET surface area evolution with grinding duration.

XRD analysis showed that CaCO_3_ did not transform to another crystalline phase during wet milling. Specifically, the diffractogram of untreated and wet‐milled CaCO_3_ contained the characteristic reflections of calcite at 2*θ* = 23.0°, 29.4°, 31.4°, 35.9°, 39.4°, 43.2°, 47.1°, 47.5°, 48.5°, 56.6°, and 57.4° (**Figure** **5**a). No other diffractions were observed even after 480 min of wet milling. This is in contrast to other studies of dry milling of CaCO_3_,^[^ ^29^, ^30^, ^31^ ^]^ where a phase transition to the high‐pressure phase, aragonite, was observed. It is assumed that the presence of water within the process distributes the impact and shear forces, leading to lower local pressures and temperatures that are insufficient for phase transformation processes.^[^ ^38^ ^]^

Isolated direct impacts or isolated shear‐forces yield different effects on the microstructure of sorbent particles as compared to a simultaneous combination of intense shear and impact forces.^[^ ^36^ ^]^ A higher sorbent CO_2_ capacity can be achieved if the preparation results in low crystallinity and small crystal size after the calcination of CaCO_3_ to CaO. The possibility of particle growth in mills is explained by transient hot spots leading to cold‐welding phenomena and plasma formation. In such transient, high‐energy‐density environments, activated domains tend to agglomerate when particles are trapped between colliding grinding bodies, which causes flattened layers to overlap and form cold welds.^[^ ^47^ ^]^ The crystallite sizes of the planetary milled sorbents used in this work decreased rapidly with increasing milling time (Figure 5b) until they reached a minimum after 30 min. However, at long milling times exceeding 60 min, the crystallite size increased again. The reduction in crystallite size in the first stage is expected, while the growth can be caused by the heat accumulation in the milling vessel, which can promote crystal growth. Similarly, the creation of defects and dislocations can lead to nucleation and consecutive crystallite growth.

A larger number of smaller grinding bodies produced small crystallites faster than bigger grinding bodies. This can be explained by the higher number of collisions inside the vessel, while the intensity of these collisions appears to be less critical.^[^ ^48^, ^49^ ^]^ For the CLP, a small crystallite size of CaO is beneficial because it results in a high density of defects and grain boundaries, facilitating mass transfer in the subsequent carbonation cycle.^[^ ^39^, ^44^ ^]^ To the best of our knowledge, there have not been any reports about CLP crystallite growth in planetary wet‐milled systems, as shown in Figure 5b. Still, it can be inferred that additional investigation might be relevant with respect to its consequences for the use of milled sorbents in the CLP.

**Figure 6:**
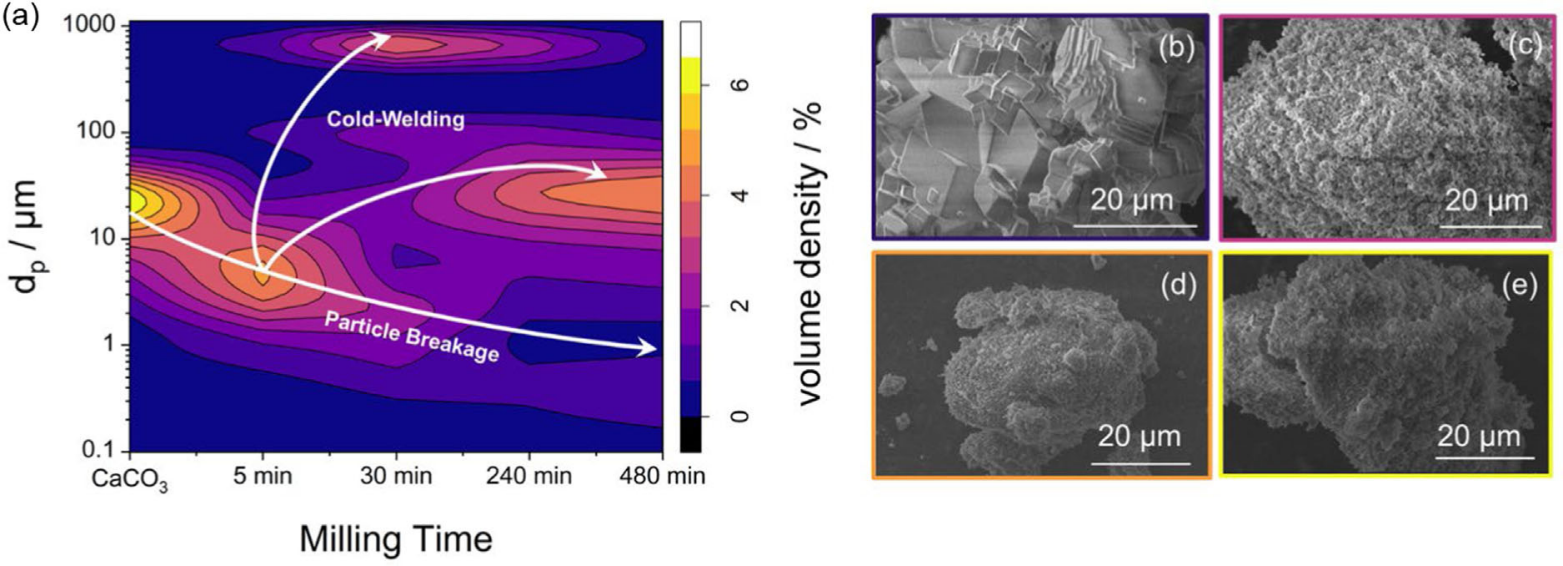
a) Contour map of particle size (d_p_) in microns as a function of milling time in minutes for 100 × 3/16” grinding bodies. The volume fraction is denoted by color from 0% (dark purple) to 5.9% (light yellow). Qualitative trends are similar for 7/16” grinding bodies. b–e) SEM micrographs of untreated (a), 30 min (b), 240 min (c), and 480 min (d) wet‐milled and dried CaCO_3_. Long milling shows an agglomeration of small particles on the surface of larger ones, resulting in particle size growth.

Besides the crystallite size, the particle size (if not adjusted, e.g., via pelletization) has been reported to have a strong influence on the reaction rate and process efficiency, which can be reasoned by the smaller diffusional resistance as well as a higher accessible surface.^[^ ^11^ ^]^ PSDs showed an original size of d_50_ = 18.4 μm and a unimodal distribution for untreated CaCO_3_ (**Figure** **6**a, summarizing all individual PSD determinations). With milling, the particle size was first reduced to a minimal value of d_50_ = 3.9 μm after 30 min. At the same time, the distribution changed to a bimodal configuration. With increasing milling duration, the PSD shifted back to larger particles, e.g., d_50_ = 20.8 μm after 480 min. This was already observed in dry‐grinding applications and is associated with the cold‐welding of particles in mechanochemical systems.^[^ ^36^ ^]^ The change of regimes from breakage to back growth is explained by the kinetics of the milling treatment. First, a high concentration of large particles favors breakage. After passing a minimum, the effects of the slower cold‐welding become more pronounced until the equilibrium is reached, as indicated by the nearly identical PSDs for 240 and 480 min. Particle growth occurs more readily for softer materials such as calcite (Mohs hardness: 3).^[^ ^50^ ^]^

The morphological evaluation of the samples through SEM displayed the production of high surface roughness with increasing milling time. The initial, ordered structure was fractured into smaller particles in the first 30 min and later agglomerated (Figure 6b–e). Therefore, milling conditions have to be chosen carefully. During the CO_2_ adsorption cycle, little shavings caused by abrasion agglomerated around bigger particles and influenced the sorbent capacity. It is reasonable to suggest that the resulting increase in the surface‐to‐volume ratio will accelerate CO_2_ diffusion by reducing the diffusion length to the unconverted CaO site. If these shavings become very small and group around bigger particles, this can become a drawback. The tendency of the shavings to sinter faster can reverse the benefits of the originally increased surface area. The explanation lies in the exclusion of a major CaO fraction to react within the FRP. This is attributed to the fast formation of an outer CaCO_3_ shell originating from these shavings. As soon as it exceeds the critical diffusion layer thickness, it will seal off unconverted CaO, transferring the system to the SDP regime earlier.

The change in morphology was confirmed by the porosity of the samples. The recorded Type II‐like isotherms illustrated the meso‐/macroporosity of CaCO_3_^[^ ^51^ ^]^ (Figure S5, Supporting Information). With increasing milling duration, the surface area values of uncalcined sorbent initially increased and leveled off with sufficient milling time, e.g., 21.2 m^2^ g^−1^ after 480 min of milling with 100 × 3/16″ grinding bodies (Figure 5c).

**Figure 7:**
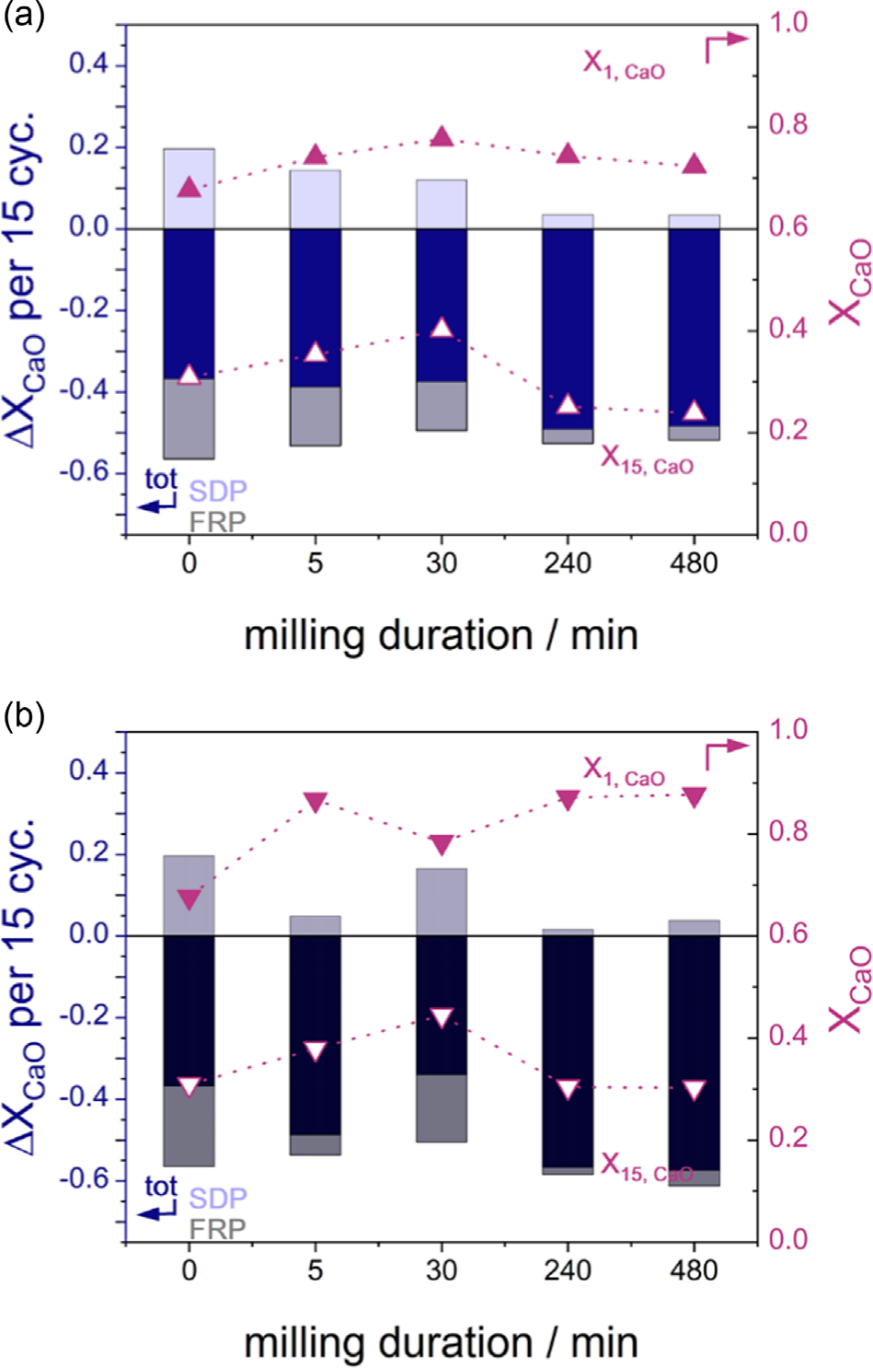
CaO conversion for the first (filled triangles) and 15th (open triangles) cycle for planetary wet‐milled CaCO3 with a) 8 × 7/16″ and b) 100 × 3/16″ grinding bodies (right axis). Total change of conversion (dark shading) compared to the first cycle with the respective contributions of the FRP (medium shading) and SDP (light shading) (left axis). 0 min represents the as‐received, untreated CaCO_3_.

The multicyclic CaO conversion, X_N_, reflected the consequences of the sorbent properties for the CO_2_ uptake within the CLP. For the initially planetary wet‐milled sorbent, samples with intermediate milling durations showed the highest cumulative sorbent capacity over 15 cycles for both FRP and SDP (**Figure** **7**). In general, a larger number of smaller balls seemed to favor the formation of a high‐capacity sorbent compared to a smaller number of bigger grinding bodies. To highlight the reasons for this, we can compare the total CO_2_ uptake for sorbents prepared with different numbers of grinding bodies, but similar characteristics (e.g., same surface area, crystallite size, morphology). This is the case for CaCO_3_ samples milled for 15 min with a hundred 3/16″ balls and 120 min with eight 7/16″ balls, respectively. The overall CO_2_ uptake for these samples was similar (6.03 *g*_CO2_/*g*_sorbent_ compared to 6.14 g_CO2_·g_sorbent_^−1^). Therefore, it can be concluded that a longer milling time and, therefore, higher energy consumption, are required to prepare a sorbent with similar characteristics. The results align with the implemented model (**Table** **3**). The retrieved fitting parameter for the sintering influence (*k*_p_) suggests that short milling decreases deactivation through sintering and calcination/carbonation cycles, whereas longer milling seems to do the opposite. The choice of ball‐to‐powder ratio (which was 12.3 with 3/16″ balls and 12.6 with 7/16″ balls, respectively) was less significant. The deciding factor is rather the extent to which there is friction between grinding bodies or grinding bodies with walls. In addition, the FRP CO_2_ uptake in the first cycle aligned with the physisorption data for CaCO_3_ milled using the 3/16″ grinding bodies. However, this correlation was not observed for the sample milled with the larger 7/16″ grinding bodies, suggesting that milling conditions significantly influence the material's conversion behavior. For those samples, the physisorption results suggested that the FRP CO_2_ uptake might not differ as much since their surface area values were in a closer range.

**Table 3:** Fitting parameters of Equation ((4), (5))–(6), (12) to the data used for Figure 6.

N_gb_ [#]	Milling duration [min]	*ε*_0_ [m^3^_void_ m^−3^]	k_p_ [min^−1^]	k_m_ [min^−1^]	R^2^ [−]	*ω* ^2^ [−]
–	0	0.4	0.3	–	0.978	0.965
8	5	0.4	0.2	0.1	0.984	0.975
8	30	0.4	0.2	0.1	0.989	0.983
8	240	0.4	0.3	0.1	0.993	0.989
8	480	0.4	0.3	0.2	0.983	0.974
100	5	0.4	0.2	0.1	0.983	0.974
100	30	0.4	0.2	0.1	0.995	0.992
100	240	0.4	0.3	0.1	0.988	0.981
100	480	0.4	0.3	0.1	0.996	0.994

For initial mechanochemical activation of fresh CaCO_3_, the optimum was a treatment with 100 grinding bodies and a milling time of 30 min, as determined in Figure 6. An optimum at 30 min was also observed for the larger grinding bodies, but to a lesser extent. The presented model parameter results were consistent with this. The interconnection of XRD, TGA, PSD, and physisorption data suggested that the mechanical preparation of the sorbent not only relies on the already reported milling mechanism,^[^ ^36^ ^]^ the presence and quantity of grinding assistant, or the initial composition of the sorbent,^[^ ^38^ ^]^ but also on the energy input and, most importantly, the duration of the treatment. The SDP became more prominent for all samples over time, which is in accordance with the anticipated sintering of the sorbent. For remilling conditions, this means that the milling time should be kept as high as necessary to crack sintered structures while ensuring not to exceed an energy input leading to fusion by cold‐welding.

## 3 Conclusion

This study highlights that remilling, i.e., the milling of the sorbent after every cycle of calcination and carbonation, prolongs the sorbent lifetime for the CLP. Moreover, it multiplies the multicyclic CO_2_ uptake compared to reference samples that were initially milled, i.e., milled only once, or untreated. The sorption capacity increases because aged sorbent structures crack and reopen with every new milling cycle, thus increasing the accessibility of CaO. Milling also promotes the formation of beneficial Ca(OH)_2_, which causes microfractures, because it is less dense than CaO. This does not hinder the reaction, because Ca(OH)_2_ converts to CaO under reaction conditions. These insights were gained from characterization of the remilled CaO, which showed increased porosity, roughened surface morphology, microchannel formation, and enhanced bulk diffusion compared to the initially milled or untreated sorbent.

We identify three key parameters that need to be controlled for in each experiment: crystallite size, surface area, and hydration level. First, milling reduces the crystallite size, and smaller crystallites sinter faster. Thus, the high surface area of freshly milled material becomes inaccessible fast. Second, postmilling pelletization for the adjustment of particle size also reduces surface accessibility. Both observations underline that the contribution of remilling lies with breaking apart aged sorbent and that both crystallite size and surface accessibility are secondary effects. Third, the hydration level affects the Ca(OH)_2_ concentration of the sorbent, which, to a degree, also enhances its capacity. We show that wet remilling is twice as effective as simple sorbent hydration. This demonstrates that wet milling not only increases hydration but also prevents agglomeration.

We have demonstrated the high effectiveness of short milling durations, and thus, the feasibility of high space‐time yields in an industrial setting. Implementation of this remilling technique could be achieved with a milling step in the middle of two interconnected fluidized bed reactors with subsequent pelletization, which could increase the CLP effectiveness and decrease the replacement rate of deactivated sorbent.

## 4 Experimental Section

4.1

4.1.1

##### Materials and Sorbent Preparation

Calcium carbonate (ACS reagent grade) was purchased from Sigma‐Aldrich. Mechanochemical reactivation (remilling) of the sorbent was conducted in a Retsch PM 200 planetary ball mill after every calcination–carbonation cycle. For this, a 25 mL stainless‐steel vessel was filled with 100 stainless‐steel balls of 3/16″ diameter and 3.5 g of sorbent and 2.5 g of water. The milling duration was set to 30 min at 500 rpm. After the mechanochemical treatment, the sorbent was dried overnight (105 °C). The particle size was adjusted by pelletization under 3 tons for 120 s, crushing the agglomerate, and sieving it to afford a particle size within 425 μm < x_p_ < 850 μm. Reference samples of CaCO_3_ were used for comparison. The first reference was untreated CaCO_3_, and the second one was exclusively milled before the first cycle (initially milled). In both cases, the particle size was adjusted the same way as the remilled sorbent (425 μm < x_p_ < 850 μm). Variations in the pelletization process and the use of different batches of reference material can lead to slight deviations/errors. These may stem from minor inconsistencies in the manual operation of the hydraulic press, fluctuations in laboratory humidity, and potential measurement errors. Such factors can significantly influence the first sorption cycle within the CLP and should be carefully considered when comparing different sorbent types (e.g., reference compared to remilled sorbent).

Key parameters, such as the milling duration, ball number, and grinding body size, were varied in the sample preparation to obtain more information about the milling process. As in the previous experiments, 3.5 g of CaCO_3_ was placed in 25 mL milling vessels, with the ball‐to‐powder ratio kept constant. The milling was performed under different conditions (**Table** **4**).

**Table 4:** Experimental conditions for millin process Optimization.

Series	Duration [min]	d_grinding bodies_ [“]	N_grinding bodies_ [#]	Rel. water [*g*_H2O_/*g*_CaCO3_]	Ball‐to‐powder ratio [*g*_grinding bodes_/*g*_CaCO3_]
#1	5, 30, 240, 480	3/16	100	1.43	12.4
#2	5, 30, 240, 480	7/16	8	1.43	12.4

##### CO_*2*_ Sorption Performance

To investigate the multicycle sorption behavior of CaO, CO_2_ breakthrough curves were recorded on an Omnistar mass spectrometer from Pfeiffer Vacuum Technology AG (Supporting Information) and by TGA, which was carried out on a STA 449 F3 Jupiter from NETZSCH GmbH. The multicyclic CaO conversion defines the mass of CaO that is converted into CaCO_3_ in the carbonation stage of every single cycle (Equation (2)).

(2) $$X_{i}=\frac{\left(m_{\text{max,i}}{\text{ − m}}_{\text{min,i}}\right)\cdot M_{\text{CaO}}}{m_{\text{min,i}}\cdot M_{{\text{CO}}_{2}}}\;\left[\%\right]$$

For the analysis of recorded sorption data, the well‐known distinction between the FRP (I) and the SDP (II) for the multicyclic CaO conversion was made.^[^ ^24^ ^]^ The two phases are distinguished by the reaction rate. The FRP is characterized as the fast, first stage, followed by a kink in the mass evolution over time, characterizing the beginning of the SDP.

For remilled sorbents as well as the references, calcination and subsequent carbonation were carried out in a vertically arranged stainless‐steel fixed bed reactor with 1/2" inner diameter and a length of 55 cm. The tube was installed inside an Applied Test Systems Inc. furnace (series: 3110). Typically, 7.6 g of the milled and particle size‐adjusted sorbent was transferred into the fixed bed. The sample was heated from room temperature to 950 °C at a rate of 10 K min^−1^ and held at this calcination temperature for 1 h. Subsequently, the sample was cooled to 650 °C at a rate of 6.6 K**·**min^−1^ and held at the carbonation temperature for 4 h. For every run, a breakthrough curve was collected on an OmnistarTM mass spectrometer from Pfeiffer Vacuum Technology AG. Calcination was performed under N_2_ atmosphere and carbonation under 15 vol.‐% CO_2_ (N_2_ balanced).

**Figure 8:**
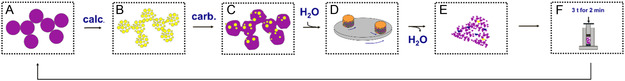
Schematic description of the remilling process steps (purple: CaCO_3_, yellow: CaO, blue: Ca(OH)_2_). Description: A) pristine CaCO_3_; B) CaO after CaCO_3_, calcination; C) CaO fragments encapsulated by a CaCO_3_ shell after sorbent carbonation; D) reactivation through wet planetary ball‐milling; E) milling products; and F) particle size adjustment.

After finalizing the temperature program, the sequence of milling, pelletizing, and calcination/carbonation was repeated 10 times. After every cycle, 100 mg of the sample was withdrawn for further characterization. Material loss was balanced through a two‐batch approach. Both batches of 7.6 g initial mass were subjected to the same reaction and milling conditions. The second batch of material was used to adjust the first batch to 7.6 g before every reaction in the fixed bed reactor. A schematic illustration of the process steps is shown in **Figure** **8**.

Reference data were collected on the same setup and under the same reaction conditions for fresh, pelletized CaCO_3_ (425 μm < x_p_ < 850 μm, 3 t for 120 s) and initially milled and pelletized CaCO_3_ (425 μm < x_p_ < 850 μm, 3 t for 120 s).

In addition, thermogravimetric analysis was carried out on a STA 449 F3 Jupiter. The temperature programs consisted of an initial calcination to remove remaining CO_2_ or other impurities, followed by carbonation‐calcination cycles. The reaction conditions were varied according to the experimental requirements (**Table** **5**).

**Table 5:** Experimental conditions for TGA experiments.

Information	Remilling	Efficiency investigation
Sample [mg]	10 ± 1	20 ± 1
Crucible material	Al_2_O_3_	Al_2_O_3_
Initial calcination	950 °C, 1 h	950 °C, 1 h
Carbonation	650 °C, 15 vol% CO_2_	650 °C, 15 vol% CO_2_
Calcination	950 °C, 1 h	900 °C, 30 min
Cycles	Fixed‐Bed Reactor	15 cycles, 30 min each
Heating rate [K min^−1^]	15 K min^−1^	15 K min^−1^
Cooling rate [K min^−1^]	40 K min^−1^	40 K min^−1^

##### Sorbent Characterization

Sorbent characterization was carried out for both sorbent types appearing during cycling (CaCO_3_ and CaO). For CaO, calcination prior to analysis was required. It was carried out in a tube furnace from Nabertherm (RS80/300/11) under a nitrogen atmosphere. The residence time within the tube furnace was 1 h (conditions chosen to mimic the desorption inside the fixed bed reactor). The calcined sorbent was stored under argon to prevent the formation of Ca(OH)_2_ through reaction with ambient humidity.

LDA was carried out on a Mastersizer 3000 E from Malvern Panalytical. The sample was prepared by sieving out oversized particles through a mesh of 212 μm. After equilibration and subtraction of background signals, a powder fraction was dispersed in a 500 mL beaker of deionized water until obscuration values were in a specified range (5%–20%). The stirrer was set to 1250 rpm, and ultrasound sonication was used for 60 s to aid breakage of loosely bound agglomerates.

Nitrogen physisorption at −196 °C was performed on an ASAP2020 from Micromeritics Instrument Corporation. Degassing was performed at 350 °C for 240 min under vacuum. The collected data were analyzed by BET surface area determination.^[^ ^52^ ^]^ Pore size distributions were calculated by applying the BJH method to the desorption branch of the adsorption isotherm.^[^ ^53^ ^]^ Cylindrical pores were assumed.

SEM images were collected on a Thermo Scientific Axia ChemiSEM. Particles of the sample were adhered by the use of carbon tape, and loose sample particles were removed by blowing air. The operation voltage was set to 5.00 kV, and an Everhart–Thornley detector was used.

Powder XRD patterns were collected on a Rigaku MiniFlex benchtop XRD diffractometer using Cu Kα radiation (*λ* = 1.5406 Å). Data were collected in a 2*θ* range of 10° to 90° with a scan speed of 0.1°/s and a step size of 0.01°. Crystallite sizes were calculated through application of the Scherrer equation^[^ ^54^ ^]^ (Equation (3)). For this, an in‐house Python code was written and applied to the main individual reflections.

(3) $$\tau =\frac{K\cdot \lambda }{\beta \cdot \text{cos}\left(\theta \right)}$$

*τ* indicates the average size of crystalline domains (nm), K is the dimensionless shape factor, *λ* refers to the wavelength (nm), *β* is the full width at half maximum of the diffraction peak (dimensionless), and *θ* represents the Bragg angle (° or rad).

The inaccuracy of the respective sorbent characterization method was determined by repeated measurement of the pristine sorbent. The estimated experimental error for d_50_ values determined by LDA, XCaO given by TGA chemisorption, N_2_ physisorption derived BET surface area, and XRD determined crystallite size were ±1.51%, ±2.15%, ±1.04%, and ±1.17% respectively.

##### Model Description

A porosity‐dependent system description was developed to compare the CO_2_ removal efficiency of in different ways processed sorbents. A base model inspired by Scaltsoyiannes et al.^[^ ^55^ ^]^ was extended. Within the framework, the CaO conversion of the FRP is correlated to the available porosity of the calcined sorbent before carbonation (Equation (4)).

(4) $$X_{i,\;\text{FRP}}=\frac{\varepsilon_{\text{CaO}}\cdot M_{\text{CaO}}}{\rho_{\text{CaO}}\cdot \left(\text{1 ‐ }\varepsilon_{\text{CaO}}\right)\cdot (V_{{M,\text{CaCO}}_{3}}\;‐\;V_{M,\text{CaO}})}$$

*X*_i,FRP_ represents the carbonation conversion during the fast reaction phase (%), *ε*_CaO_ denotes the available porosity (%), *M*_CaO_ is the molar mass of CaO (56 g mol^−^^1^), *ρ*_CaO_ refers to the density of CaO (3.35 g cm^−^^3^), *V*_M,CaCO__3_ is the molar volume of CaCO_3_ (36.9 cm^3^ mol^−^^1^), and *V*_M,CaO_ is the molar volume of CaO (16.7 cm^3^ mol^−^^1^).

As mentioned in their work, similar approaches were already used in multiple other investigations.^[^ ^56^, ^57^, ^58^ ^]^ CaO porosity is changing while the sorbent is cycled in the process. Since sintering is one of the main reasons for the loss of sorbent capacity by reducing porosity and closing of accessible surface, the relationship proposed by Coble^[^ ^59^ ^]^ is used to model this temperature‐induced and time‐dependent effect (Equation (5)).

(5) $$\varepsilon (\text{t,T})=\frac{\varepsilon_{0}}{k_{p}\cdot \text{ln}\left(\frac{t}{t_{c}}\right)}$$

The available porosity, denoted as *ε*(t,T), varies with time and temperature and is expressed as a percentage. The initial available porosity is represented by *ε*_0_, also in percent. The sintering process is characterized by the sintering constant (*k*_p_), a dimensionless parameter. The total sintering duration (t) and the induction period (*t*_c_)—both measured in minutes—are time variables that govern porosity evolution during sintering.

However, this parametric description is insufficient to capture the effect of milling. Therefore, a time‐dependent milling term is implemented into the previous set of equations. Since milling is dependent on various factors, and to simplify the model, a straightforward first‐order breakage term is used to describe its time dependency.^[^ ^60^ ^]^ The given equation correlates the porosity of the sorbent with the duration of milling (Equation (6)). As can be seen, an increase in milling duration yields a sorbent of higher porosity. This assumption is only valid upon certain restrictions, for example, a porosity within the percentage range (0, 100). In consequence, the milling rate constant will reflect the effectiveness of the procedure, but also the appearance of mill‐induced sintering, e.g., also for activated surfaces.

(6) $$\varepsilon \left(\text{t,I}\right){=\varepsilon }_{0}\cdot {\left(\frac{x_{0}}{x}\right)}^{\beta }{=\varepsilon }_{0}\cdot {\left(\frac{x_{0}}{x_{0}\cdot e^{‐k_{m}\cdot t_{m}}}\right)}^{\beta }{\text{= ε}}_{0}\cdot {\text{ e}}^{\beta \cdot k_{m}\cdot t_{m}}$$

*ε*(t,I) denotes the available porosity as a function of time and intensity (%), *ε*_0_ is the initial available porosity (%), *x* represents the particle size (nm), x_0_ is the initial particle size (nm), β is a fitting parameter (dimensionless), *k*_m_ is the milling rate constant (min^−^^1^), and *t*_m_ is the milling duration (min).

Another aspect to be considered within the conversion calculations is the dispersion of the sorbent in water while milling. As soon as the first calcination is finished, residues of CaO surrounded by CaCO_3_ shells will be present. The reaction between CaO and H_2_O can cause a density change from 3.35 g cm^−^^3^ (CaO) to 2.24 g cm^−^^3^ (Ca(OH)_2_).^[^ ^20^ ^]^ This swelling effect is considered the major origin of fine, small‐scale cracks (Equation (7)).

(7) $$\Delta V=V_{{\text{Ca(OH)}}_{2}}\text{ ‐ }V_{\text{CaO}}$$

Similarly, water trapped in pores might leave as steam during drying or calcination, causing pressure buildup that can rupture pore walls and also create cracks if it surpasses the system's tension limit. In consequence, a time‐dependent relationship for the mill treatment and the porosity introduced by CaO‐Ca(OH)_2_ interconversion is suggested for the present model. Again, a first‐order approach is used to model this behavior (Equation (8)). To account for the volume increase introduced by small cracks, one additional fitting parameter, *V*_c_, is proposed. The milling duration is defined as before, while the kinetic constant is a summarizing parameter accounting for the closure or opening of activated surfaces and microcracks.

(8) $$\varepsilon \left(\text{t,I, }x_{\text{CaO}}\right){=\varepsilon }_{0}+\frac{V_{C}\cdot \left({\text{1‐ e}}^{{\text{‐k}}_{\text{Ca}{\left(\text{OH}\right)}_{2}}\cdot \;t_{m}}\right)}{V_{M,\text{CaO}}}$$

*ε*(t,I,x_CaO_) represents the available porosity as a function of time, intensity, and the CaO mass fraction in the sorbent (%), *V*_C_ is the microcrack volume resulting from Ca(OH)_2_ swelling (cm^3^), *k*_Ca(OH)__2_ is the rate constant for Ca(OH)_2_ formation within the mill (min^−^^1^), and *t*_m_ denotes the milling duration (min).

Summarizing, all treatment steps are described in a time‐dependent manner. Therefore, the residence time of all steps is fed to the model in a hardcoded way. Within the carbonation and calcination cycles, only sintering and calcination+carbonation‐induced deactivation occurs. This leads to the following calculation procedures, where 2) and 3) are extended for milling and Ca(OH)_2_ formation:

1) Untreated sorbent

(9) Calcination+Carbonation→Calcination+Carbonation→…

2) Initially milled sorbent

(10) Milling→Calcination+Carbonation→Calcination+Carbonation→…

3) Remilled sorbent

(11) $$\text{Milling}\to \text{Calcination}+\text{Carbonation}\to \text{Milling}+\text{Ca}{\left(\text{OH}\right)}_{2}\text{formation}\to \text{Calcination}+\text{Carbonation}\to \text{Milling}+\text{Ca}{\left(\text{OH}\right)}_{2}\text{formation}\to \ldots$$

As soon as all changes of porosity for a specific treatment have been covered, the resulting porosity for the next cycle i can be calculated according to Equation (12).

(12) $$\varepsilon_{i\;}=\varepsilon \left(t\right)\cdot \frac{X_{\text{tot, FRP, i‐1}}\cdot {\text{ V}}_{{\text{M,CaCO}}_{3}}}{\left({\text{1‐X}}_{\text{tot, FRP, i‐1}}\right)\cdot V_{\text{M,CaO}}{\text{ + X}}_{\text{tot,FRP, i‐1}}\cdot V_{{\text{M,CaCO}}_{3}}\;}$$

*ε*_i_ represents the available porosity for cycle *i* (%), and *ε*(t) is the time‐dependent available porosity, based on previous porosity calculations (%). Parameterization is independently carried out for the different procedures. Parameter bounds were set identically for all experiments, and constants were applied similarly to all processes. An in‐house Python code was written, and the nonlinear least‐squares problem with bounds on the variables was solved using scipy.optimize.least_squares.

##### Energy Demand Comparison

To evaluate the feasibility and sustainability of mechanochemical reactivation after every CLP cycle, this work compares the process energy demand for three distinct scenarios: i) Remilling‐enhanced CLP. ii) Regular CLP with sorbent replacement (usual procedure). iii) Regular CLP with sorbent replacement (usual procedure), including transportation of fresh sorbent to the process site.

As all scenarios share the same core process configuration, the comparison focuses on the differences in energy demand rather than absolute totals. In scenario (i), mechanical energy is required for remilling. In scenarios (ii) and (iii), the energy cost (and therefore energy demand difference) stems from the embodied energy of fresh sorbent, including its prior processing (including milling) and, in (3), its transport.

Laboratory results indicate a FRP plateau in residual CaO conversion at *X*_CaO, FRP_ = 0.35, after repeated cycling. This was used to conservatively estimate the proportion of sorbent contributing to CO_2_ capture. In contrast to this rather pessimistic scenario, an optimistic scenario of *X*_res_ = 0.5 was chosen to highlight the strength of remilling upon optimization and anticipated efficiency increase with scale‐up.

The average sorbent conversion for CLP with replacement was calculated as

(13) $$X_{\text{ave, CLP}}=\left[\frac{f\left(\text{1‐b}\right)f_{p}}{f_{p}\text{ + 1‐f}}\text{ + b}\right]$$

with *f* = 0.77, *b* = 0.17, and *f*_p_ as the purge fraction, based on Romeo et al.^[^ ^61^ ^]^ The fraction of sorbent heated within the calcination step without contributing to CO_2_ capture (herein treated as sealed off CaO) was considered as an energy penalty

(14) $$q_{\text{cc}}\text{ =}\frac{Q}{m_{\text{CaO}}}=\int_{T_{1}}^{T_{2}}c_{p}\left(T\right)\text{dT}$$

The temperature‐dependent heat capacity of CaO was taken from NIST‐JANAF Thermochemical Tables^[^ ^62^ ^]^ using

(15) $$c_{p}\left[\frac{J}{\text{mol}\cdot K}\right]=A+B\cdot t+C\cdot t^{2}+D\cdot t^{3}+\frac{E}{t^{2}}$$

(16) $$q_{\text{pen, CLP}}=f_{\text{sorbent, unused}}\cdot q_{\text{cc}}$$

with coefficients *A* = 49.95403, *B* = 4.887916, *C* = –0.352056, *D* = 0.046187, and *E* = –0.825097.

Normalization per mol of CO_2_ captured

(17) $$q_{{\text{pen, CO}}_{2}}=\frac{q_{\text{pen, CLP}}}{X_{\text{CaO}}}$$

Normalization was conducted similarly to account for milling or transportation of the sorbent. Integration was performed between 650 °C (carbonation) and 850–1150 °C (calcination). Lower calcination temperatures (e.g., 700 °C), while often used in lab studies, were excluded due to their incompatibility with industrial‐scale CLP requirements (high CO_2_ partial pressure).

For comparability with industrial practice, energy input for sorbent grinding was estimated based on a typical industrial ball mill. This is often a rotating cylindrical drum partially filled with grinding media and grinding bodies.^[^ ^63^ ^]^ Although the laboratory experiments used a planetary mill, characterized by higher specific energy demand and different grinding dynamics, a conservative lower‐bound estimate of the specific energy demand for planetary milling is reported within the results to contextualize the findings and compare lab‐ and large‐scale.

To approximate the industrial scale, analogies were used, and energy data for limestone grinding (lower bound: ≈0.4 kJ mol_sorbent_^−^^1^) and transport (≈17.34 kJ mol_sorbent_^−^^1^ per 100 km by road; 2.22 kJ mol_sorbent_^−^^1^ per 100 km by rail) were adopted from de Marco et al.^[^ ^64^ ^]^ This is justified via the similarity between limestone and CaCO_3_.

While a full LCA is beyond this work's scope, this comparison provides a first estimate of remilling's energy implications at scale. Notably, even fresh sorbent requires prior comminution, meaning mechanical energy input is relevant in all cases, with the extent depending on the replacement rate and average conversion.

## Conflict of Interest

The authors declare no conflict of interest.

## Supporting information

Supplementary Material

## Data Availability

The data that support the findings of this study are available from the corresponding author upon reasonable request.
