# Supplementary material for: Process Enhancement of Calcium Looping through Combined Mechanical and Chemical Sorbent Reactivation
Source: ChemSusChem. 2025 Aug 13;18(19):e202500835. doi: 10.1002/cssc.202500835 (PMC12487757; doi:10.1002/cssc.202500835)
Supplement: Supplementary file 1 — Supplementary Material [file CSSC-18-e202500835-s001.pdf]

Supplementary Information for

Process Enhancement of Calcium Looping through Combined Mechanical and Chemical Sorbent Reactivation

Dominik Groh,[a, b] Pawel Chmielniak,[a] Christopher W. Jones,*,[a] and Carsten Sievers *,[a]

[a] D. Groh, Dr. P. Chmielniak, Dr. C.W. Jones, Dr. C. Sievers

School of Chemical & Biomolecular Engineering
Georgia Institute of Technology
311 Ferst Dr, Atlanta, GA 30332, United States
E-mail: christopher.jones@chbe.gatech.edu; carsten.sievers@chbe.gatech.edu

[b] D. Groh
Department of Chemical and Bioengineering
Friedrich-Alexander Universität Erlangen-Nürnberg
Cauerstr. 4, 91058 Erlangen, Germany

This file includes:

Figs. S1 to S7

WILEY-VCH

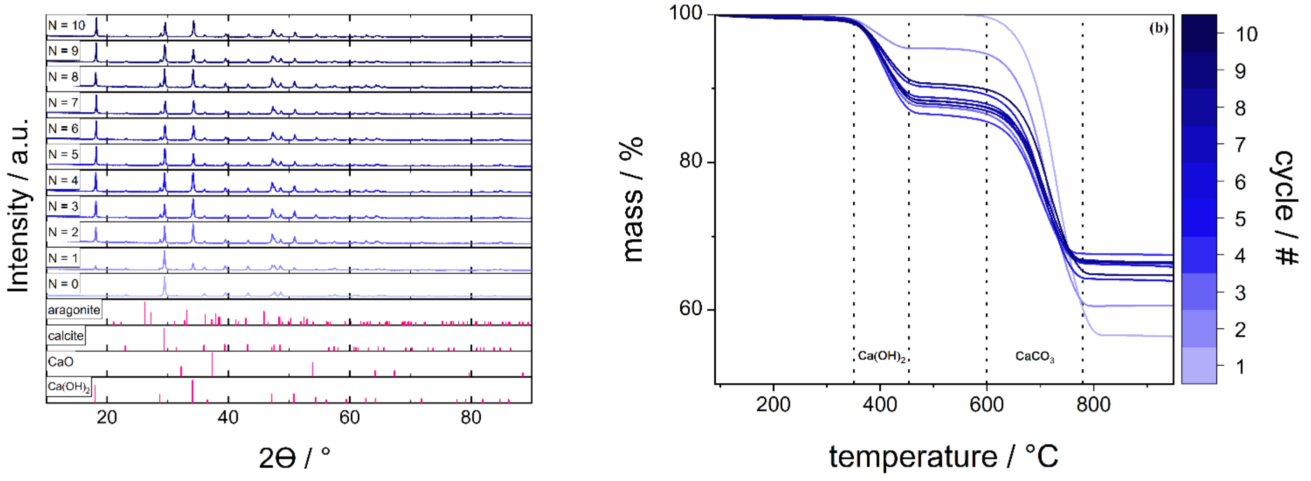

Figure S1: Decomposition behavior of remilled sorbent indicating the formation of Ca(OH)2 for N > 2 (a). Diffractograms confirm the findings of $Ca(OH)_2$ formation for the crystalline portion of the sorbent (b).

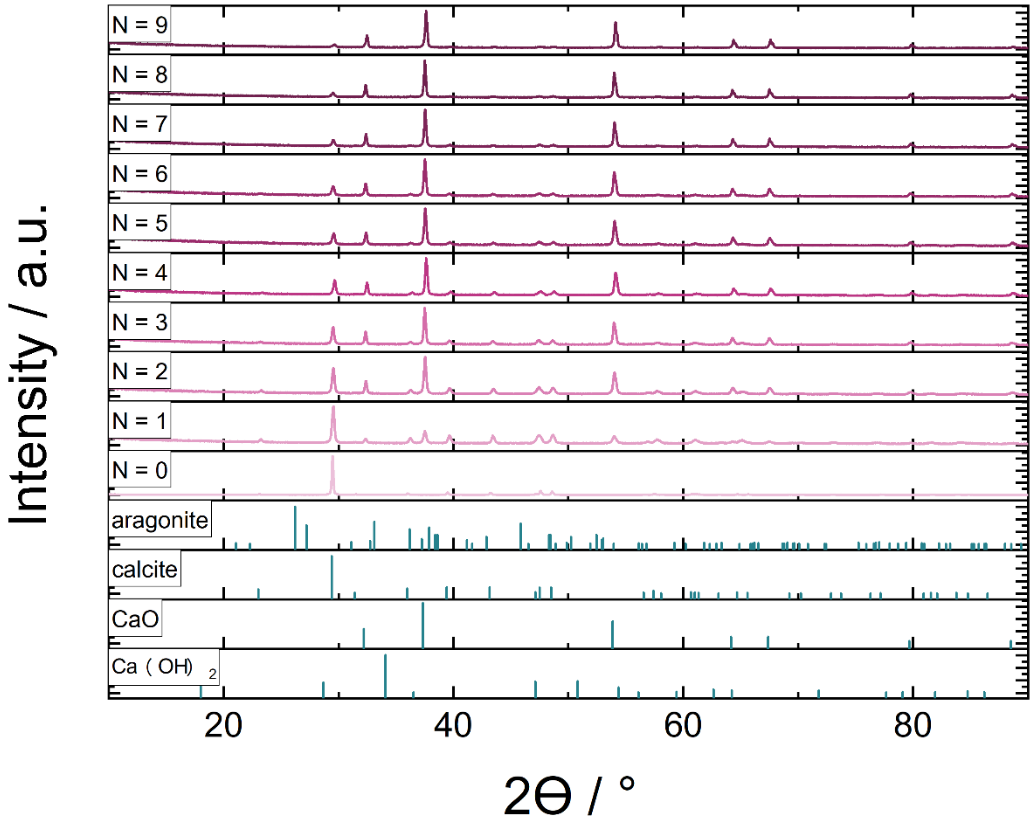

Figure S2: Diffractograms confirm calcite as only crystalline phase for untreated material during all cycles.

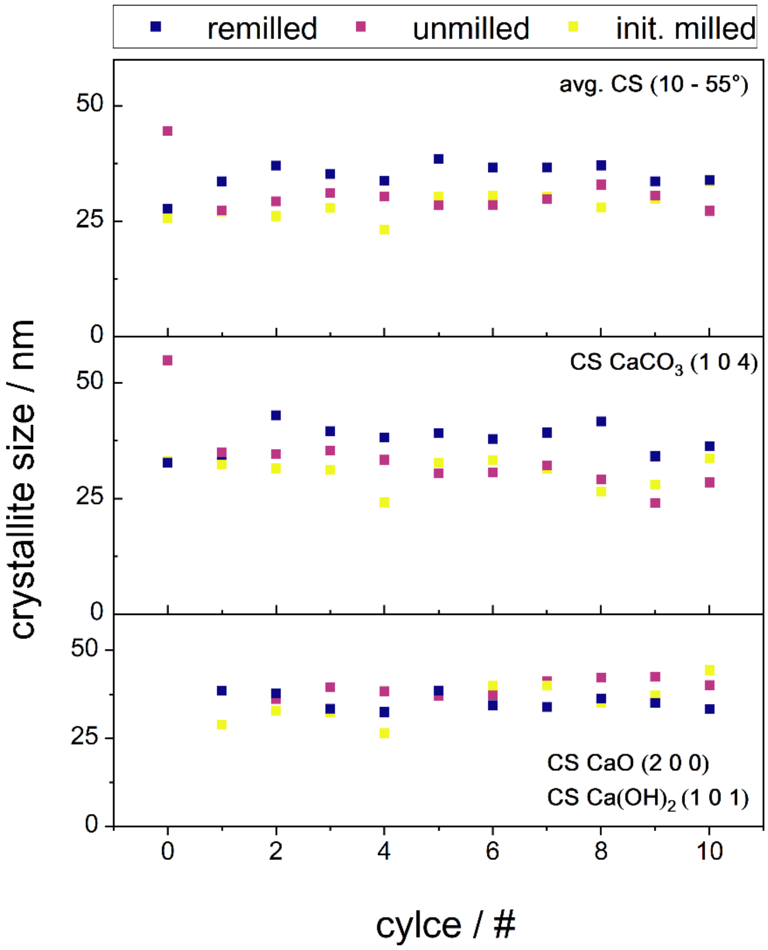

Figure S3: Monitoring of the crystal size for remilled (blue), untreated (red) and initially milled (yellow) CLP sorbent.

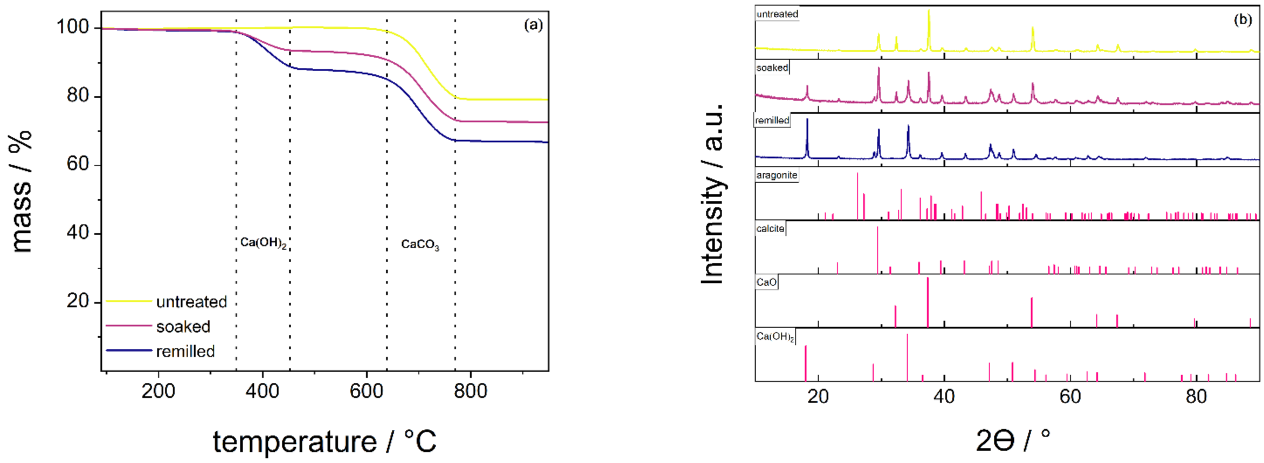

Figure S4: Decomposition behavior of untreated (yellow), water soaked (red), and remilled (blue) sorbent (a). XRD diffractograms are displayed for the same samples (b) and show highest formation of $Ca(OH)_2$ for wet remilled sorbent ((1 0 1) facet).

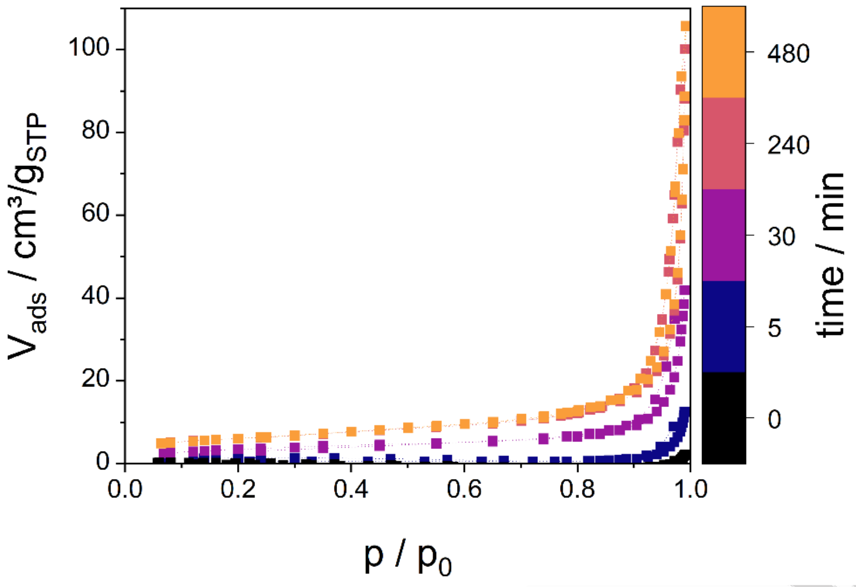

Figure S5: N_2 physisorption isotherms for different milling durations (100 x 3/16” grinding bodies).

WILEY-VCH

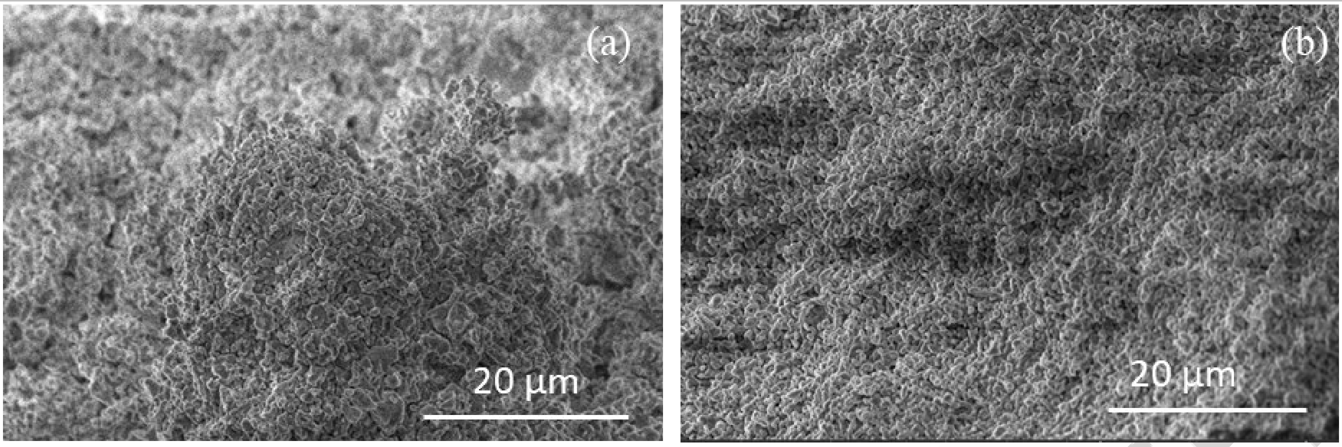

Figure S6: SEM micrographs of 30 minutes (a) and 60 minutes (b) planetary wet milled samples after 15 cycles of carbonation/calcination.

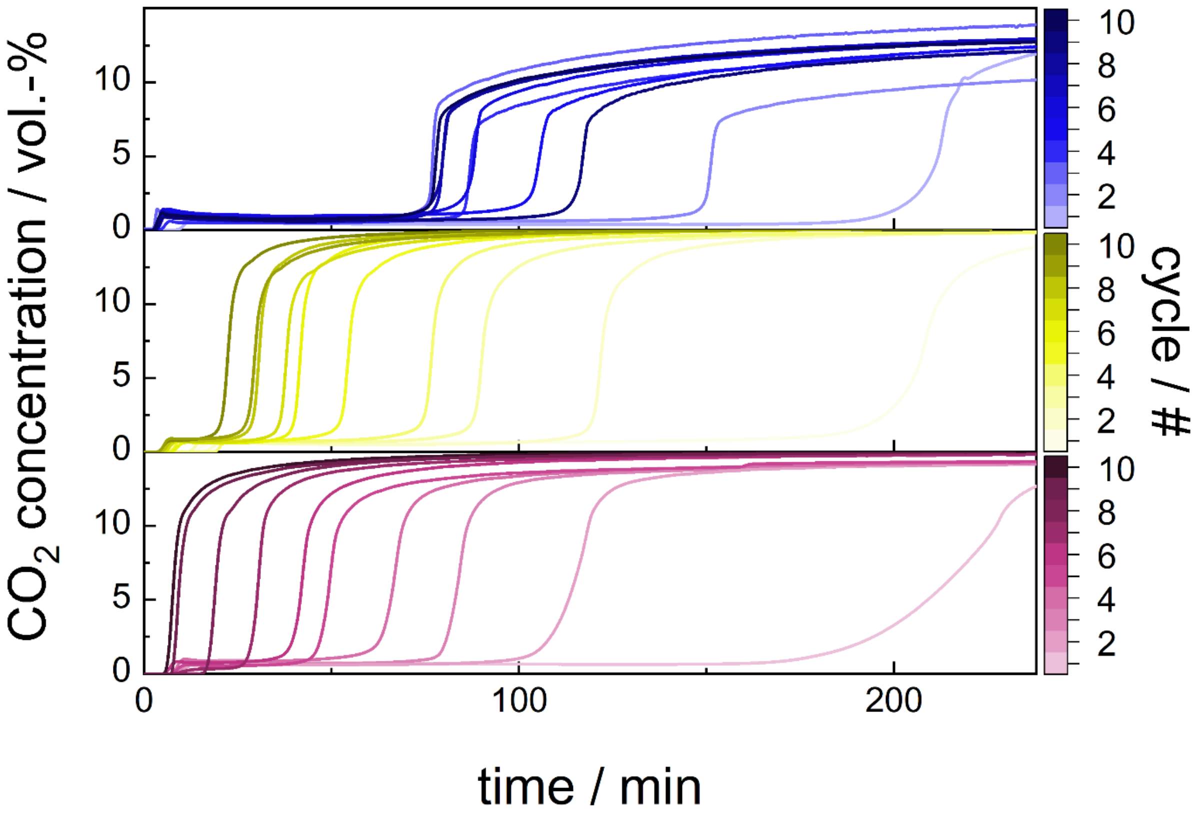

Figure S7: Breakthrough curves for remilled, untreated, and initially milled sorbent.
